# Assessment of bone marrow-derived Cellular Therapy in progressive Multiple Sclerosis (ACTiMuS): study protocol for a randomised controlled trial

**DOI:** 10.1186/s13063-015-0953-1

**Published:** 2015-10-14

**Authors:** Claire M. Rice, David I. Marks, Yoav Ben-Shlomo, Nikos Evangelou, Paul S. Morgan, Chris Metcalfe, Peter Walsh, Nick M. Kane, Martin G. Guttridge, Gail Miflin, Stuart Blackmore, Pamela Sarkar, Juliana Redondo, Denise Owen, David A. Cottrell, Alastair Wilkins, Neil J. Scolding

**Affiliations:** School of Clinical Sciences, Southmead Hospital, University of Bristol, Bristol, BS10 5NB UK; Bristol Institute of Clinical Neurosciences, Southmead Hospital, Bristol, BS10 5NB UK; Adult BMT Unit, Bristol Royal Hospital for Children, University Hospitals Bristol NHS Foundation Trust & University of Bristol, St Michael’s Hill, Bristol, BS2 8BJ UK; School of Social and Community Medicine, University of Bristol, Canynge Hall, 39 Whatley Road, Bristol, BS8 2PS UK; Queen’s Medical Centre, School of Medicine, University of Nottingham, Nottingham, NG7 2UH UK; NHS Blood and Transplant, North Bristol Park, Bristol, BS34 7QH UK

**Keywords:** Cellular therapy, Bone marrow, Progressive multiple sclerosis, Reparative therapy, Stem cell, Global evoked potentials

## Abstract

**Background:**

We have recently completed an evaluation of the safety and feasibility of intravenous delivery of autologous bone marrow in patients with progressive multiple sclerosis (MS). The possibility of repair was suggested by improvement in the neurophysiological secondary outcome measure seen in all participants. The current study will examine the efficacy of intravenous delivery of autologous marrow in progressive MS. Laboratory studies performed in parallel with the clinical trial will further investigate the biology of bone marrow-derived stem cell infusion in MS, including mechanisms underlying repair.

**Methods/design:**

A prospective, randomised, double-blind, placebo-controlled, stepped wedge design will be employed at a single centre (Bristol, UK). Eighty patients with progressive MS will be recruited; 60 will have secondary progressive disease (SPMS) but a subset (*n* = 20) will have primary progressive disease (PPMS). Participants will be randomised to either early or late (1 year) intravenous infusion of autologous, unfractionated bone marrow. The placebo intervention is infusion of autologous blood. The primary outcome measure is global evoked potential derived from multimodal evoked potentials. Secondary outcome measures include adverse event reporting, clinical (EDSS and MSFC) and self-assessment (MSIS-29) rating scales, optical coherence tomography (OCT) as well as brain and spine MRI. Participants will be followed up for a further year following the final intervention. Outcomes will be analysed on an intention-to-treat basis.

**Discussion:**

Assessment of bone marrow-derived Cellular Therapy in progressive Multiple Sclerosis (ACTiMuS) is the first randomised, placebo-controlled trial of non-myeloablative autologous bone marrow-derived stem cell therapy in MS. It will determine whether bone marrow cell therapy can, as was suggested by the phase I safety study, improve conduction in multiple central nervous system pathways affected in progressive MS. Furthermore, laboratory studies performed in parallel with the clinical trial will inform our understanding of the cellular pharmacodynamics of bone marrow infusion in MS patients and the mechanisms underlying cell therapy.

**Trial Registration:**

ISRCTN27232902 Registration date 11/09/2012. NCT01815632 Registration date 19/03/2013

## Background

Multiple sclerosis (MS) affects approximately 2.5 million people worldwide. Although most patients present with relapsing-remitting disease, over 80 % of patients develop progressive disability. Although treatments to reduce relapse frequency are available, very limited progress has been made in the prevention or reversal of progressive disability. The development of cell therapy to treat progressive MS is an attractive option due to the multiplicity of actions that may be employed including immunomodulatory, neuroprotective and reparative processes.

We have recently completed a phase I safety and feasibility study of intravenous autologous bone marrow (BM) infusion in patients with progressive MS [1]. This study not only confirmed safety but also raised the possibility of partial repair; conduction times in multiple central nervous system (CNS) pathways collated as a composite score known as the global evoked potential (GEP) [2, 3] improved in all patients studied (*n* = 6) [1]. We believe that this requires urgent investigation to determine if autologous bone marrow infusion does indeed exert a genuine reparative effect in progressive MS.

## Methods

### Objective and hypothesis

We hypothesise that intravenously delivered autologous bone marrow cell therapy (BMCT) in chronic MS offers significant benefit. We further postulate that the mechanisms are multiple, and include immunomodulation *and* reparative and/or neuroprotective effects within the CNS; and are offered by one or more BM stem cell sub-populations, jointly contributing to the therapeutic impact. Exploring and understanding these mechanisms, and the biology of the cells responsible, will allow the development of more effective reparative cell therapy in MS.

On this underlying hypothesis, we propose a phase II controlled trial in parallel with a significant body of translational and back-translational laboratory research, with the following objectives:

1. To determine the efficacy of intravenous infusion of autologous bone marrow cells in patients with progressive MS
2. To collect additional safety data regarding the collection and intravenous infusion of bone marrow cells in those with MS
3. The laboratory arm of the study will explore the mechanisms underlying the efficacy of infusion of bone marrow cells in MS to determine how these can be augmented and, potentially, how the need for bone marrow harvest can be obviated.

### Trial design

**Fig. 1:**
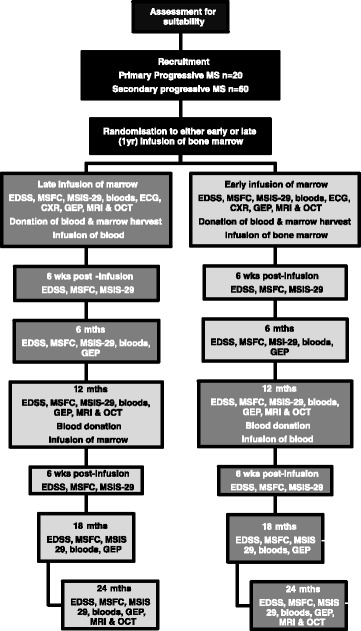
Study schema for the ACTiMuS trial

Assessment of bone marrow-derived Cellular Therapy in progressive Multiple Sclerosis (ACTiMuS) is a double blind, randomised, placebo-controlled single centre, stepped wedge design trial [4] in adults with progressive MS and Expanded Disability Status Scale (EDSS) score 4.0–6.0 (walking affected by disease, but still able to walk, with aids if necessary; distance as determined by the Ambulation Score). The study schema is presented in Fig. 1.

### Sample size estimation

We will randomly allocate 40 patients, 30 with secondary progressive disease (SPMS) and 10 with primary progressive disease (PPMS), to each arm of the study, allowing a difference of between 0.63 and 0.73 of a standardised difference in GEP between the arms to be detected with 80 % power at the 5 % significance level, assuming a loss to follow-up rate of between 0 and 25 % respectively. Our experience suggests that, given the close involvement of patients and staff, we will achieve around 95 % follow-up.

### Eligibility and enrolment

Participants will be patients attending the Bristol and Avon Multiple Sclerosis (BrAMS) Unit, North Bristol NHS Trust, UK. To enter the study, participants will have progressive MS and will have given informed consent. In addition, they must fulfil the inclusion and exclusion criteria as detailed in Table 1.

**Table 1:** Eligibility criteria for the ACTiMuS trial

Inclusion criteria	Exclusion criteria
Either sex, 18–65 years old	Pregnancy, breastfeeding or lactation
Diagnosis of clinically definite MS as defined by the McDonald criteria	History of autologous/allogeneic bone marrow transplantation or peripheral blood stem cell transplant
	Bone marrow insufficiency
MS disease severity EDSS 4–6	History of lymphoproliferative disease or previous total lymphoid irradiation
	Immune deficiency
Disease duration >5 years	History of current or recent (<5 years) malignancy
Disease progression (not attributable to relapse) in the year prior to entry	Chronic or frequent drug-resistant bacterial infections or presence of active infection requiring antimicrobial treatment
Signed, written informed consent	Frequent and/or serious viral infection
Willing and able to comply with study visits according to protocol for the full study period	Systemic or invasive fungal disease within 2 years of entry to study
	Significant renal, hepatic, cardiac or respiratory dysfunction
	Contraindication to anaesthesia
	Bleeding or clotting diathesis
	Current or recent (within preceding 12 months) immunomodulatory therapy other than corticosteroid therapy
	Treatment with corticosteroids within the preceding 3 months
	Significant relapse within preceding 6 months
	Predominantly relapsing-remitting disease over preceding 12 months
	Radiation exposure in the past year other than chest/dental x-rays
	Previous claustrophobia
	The presence of any implanted metal or other contraindication to MRI
	Participation in another experimental study or treatment within previous 24 months

### Randomisation and blinding

Randomisation will be centrally allocated using the infrastructural facilities of the Bristol Randomised Trials Collaboration (Departments of Social and Community Medicine), which provides web-based automatic randomisation services. Randomisation will be stratified by disease type (PPMS versus SPMS) using a permuted block design to ensure balance of allocation to the immediate or delayed treatment groups. Allocations are only released once the new study participant is logged into the system, thus ensuring allocation concealment. The results of randomisation will be held by NHS Blood and Transplant (NHSBT) and will be released only when the trial is to be unblinded and at the request of the Trial Management Committee.

During infusion, the trial product (blood or marrow) will be shielded from the participants using covered giving sets and obscuring the cannula site from the participant. Trial assessors do not have access to information regarding the order in which blood and marrow are infused. The level of unmasking of participants, treating physicians and assessors will be assessed by direct questioning at the end of the study.

### Trial interventions

#### Venesection

Venesection of approximately 500 mL will be performed at entry and at 1 year. Blood donation will be performed in accordance with NHS Blood and Transplant standard operating procedures which comply with UK guidelines [5]. Briefly, a cannula is inserted in the antecubital fossa and venesection is performed with a sphygmomanometer cuff inflated to 60 mmHg.

#### Bone marrow harvest and infusion

Bone marrow harvest will be performed under general anaesthesia. Approximately 500–600 mL marrow will be collected together with bone marrow trephine. The marrow aspirate will be filtered, bagged and labelled by NHSBT. An infusion of either blood or marrow will be performed on the day of bone marrow harvest. Analgaesia will be offered and a check FBC will be performed. Iron replacement therapy will be given if Hb <9.5 g/dL pre-harvest or Hb <7.5 g/dL post-harvest (ferrous sulphate 200 mg od for 1 month). One year later, intravenous infusion of blood or thawed marrow will be performed (whichever was not infused at the time of harvest).

Bone marrow trephines and a small sample of the bone marrow aspirate will be retained for back-translational laboratory research studies running in parallel with the clinical trial if specific written informed consent is given. Blood samples for research purposes may be requested throughout the duration of the study.

### Outcome measures

#### Primary outcome measure

Multimodal evoked potentials will be examined at 0, 6, 12, 18 and 24 months. Evoked potential abnormalities will be quantified according to a 4-point graded ordinal score modified from Leocani et al. (0 = normal; 1 = increased latency; 2 = increased latency and abnormal amplitude; 3 = absent) [3]. The primary outcome measure is change in GEP in the 12-month period after infusion of autologous marrow.

The recording of the evoked potentials shall be in accordance with the Guidelines of the International Federation of Clinical Neurophysiology [6], and analysis will be performed using standard methods [7] (Table 2). Electrophysiological responses shall be considered abnormal if they exceed 2.5 standard deviations of the normal values or cannot be detected.

**Table 2:** Method for recording of multimodal evoked potentials

Visual evoked potentials (VEPs) will be evoked with a rear-projected checkerboard pattern using an opto-mechanical device subtending 30 degrees at the retina, check-size 1 degree, white brightness of 150cdm^−2^ and contrast 87.5 %.
Monaural stimulation will be delivered via earphones to each side with rarefaction click stimuli of 0.1 ms duration at an intensity of 75 dB above the subjective hearing threshold whilst the contralateral ear is masked with white noise.
Sensory evoked potentials (SEPs) will be obtained by delivering electrical stimulation with square wave pulses of 0.2 ms duration to the median and the posterior tibial nerves, at the wrist and ankle respectively.
Motor evoked potentials (MEPs) will be recorded from electrodes situated over the abductor pollicis brevis muscle in the hand and the abductor hallucis in the foot using a 9 cm circular coil held over the vertex. The central motor conduction time (CMCT) will be calculated by subtracting ½(M + F + 1) from the MEP latency where M is the distal motor latency and F is the minimum F wave latency.
The GEP score will then be calculated as the sum of left and right brainstem auditory evoked potential (BSAEP) and VEP scores (0–12) and left and right upper and lower SEPs (0–12) and CMCTs (0–12).

#### Secondary outcome measures

Adverse events (AEs), serious adverse events (SAEs), safety tests, clinical measures of disability, optical coherence topography (OCT) and magnetic resonance imaging (MRI) findings are included as secondary outcome measures.

#### Adverse events

Any unfavourable and unintended sign, symptom or illness that develops or worsens during the period of the study is classified as an AE, whether or not it is considered to be related to study interventions. Adverse events include unwanted side effects, toxicity or sensitivity reactions, abnormal laboratory results and injury or intercurrent illnesses, and may be expected or unexpected.

Furthermore, if an adverse event:

- results in death
- is life threatening
- requires hospitalisation or prolongation of existing hospitalisation
- results in persistent or significant disability or incapacity
- consists of a congenital anomaly or birth defect
- or is considered by the investigator to be an important medical event,

then it is classified as an SAE and must be reported to the trial coordinating centre as soon as possible.

It is expected that some participants will be hospitalised during the study for MS-related problems. These events should be classified as SAEs and reported accordingly. However, a hospital admission for a procedure planned before entry into the study will not be recorded as an SAE.

Expected adverse events include:

- Local bruising and discomfort following bone marrow harvest
- Increase in lower limb spasticity following bone marrow harvest
- Acute urinary retention following bone marrow harvest
- Temporary exacerbation of MS following general anaesthesia
- Hypovolaemia or anaemia following blood and marrow donation
- Hypersensitivity to marrow cryopreservative
- Exacerbation of MS due to sepsis, for example, urinary tract infection or chest infection
- Assessment at or admission to hospital following fall

#### Clinical laboratory tests for safety analyses

Blood taken for safety analyses will be screened as follows: urea and electrolytes, liver function tests, full blood count with differential white cell count, coagulation, group and save, C-reactive protein, glucose, calcium, magnesium, chloride, bicarbonate, phosphate, viral serology (including cytomegalovirus, Epstein-Barr virus, herpes simplex virus, varicella zoster virus, toxoplasmosis, hepatitis B and C, human immunodeficiency virus, human T cell lymphoma virus and syphilis screening. Urinalysis (microscopy and culture) will also be performed.

#### Clinical outcome measures

Clinical outcomes will be assessed at entry and after each intervention at 6 weeks, 6 months and 1 year. The clinical rating scales will include the widely used Expanded Disability Status Scale (EDSS) [8] together with the Multiple Sclerosis Functional Composite (MSFC). The latter is a three-part quantitative assessment including a timed walk, nine-hole peg test and Paced Auditory Serial Addition Test (PASAT) [9]. In addition, participants will be asked to complete the MS Impact Scale (MSIS-29), which is a well-validated patient-completed rating scale [10–13].

#### Paraclinical outcome measures

##### Magnetic resonance imaging (MRI)

Participants will undergo cranial and spinal MRI at three time points: at baseline and at the end of years 1 and 2. The secondary MRI outcome measures will relate to 1) lesion load, 2) atrophy measures both of the brain and of cross-sectional area of the spinal cord [14], and (3) changes in mean diffusivity [15].

Exploratory analysis of the resting-state fMRI data will investigate correlations between network patterns and ‘strength’ of networks connectivity from the resting-state fMRI with classifications revealed by the various evoked potential studies [16–18].

##### Optical coherence tomography (OCT)

Measurement of retinal nerve fibre layer thickness [19] and macular volume [20] using OCT is increasingly recognized as an objective outcome measure which accurately reflects axonal loss.

The measures taken for secondary outcomes are as follows:

1. Safety: evaluation of number and nature of adverse events
2. Physician-based EDSS: time to EDSS progression of at least one point from a baseline EDSS of 4.0, 4.5 or 5.0 or at least 0.5 point from a baseline EDSS ≥5.5.
3. Patient-based MSIS-29 physical impact scale version 2: overall mean change from baseline to end of study
4. MSFC: overall mean change of z-scores, from baseline to final visit
5. MRI head: T1 weighted 3D gradient echo, 3D FLAIR, DTI and MRI cord (3D gradient echo)
6. OCT: macular volume, thickness of retinal cell layer
7. Annual overall patient and treating physician assessments of efficacy.

### Trial analyses

A full statistical analysis plan will be written prior to completion of data collection and analyses. The null hypothesis is that there will be no significant difference in the primary and secondary outcomes between intervention and control arms at (a) 12 months, and (b) 2 years. The first 12 months will be a standard comparison of the intervention against control (hypothesis a), while the assessment at 2 years will be a comparison between immediate versus delayed treatment (hypothesis b). We will also have a 2 years’ follow-up and be able to look at the longer term effects of the intervention in patients receiving it immediately.

The distribution of GEPs will be examined and, based on the prior work by Leocani and colleagues [3], it is likely that this will be relatively normally distributed or slightly skewed. In this case we will use conventional linear regression models to estimate the differences in mean GEP between study arms, with 95 % confidence intervals and *P*-values, to address the above hypotheses (a) and (b) in turn, adjusting for covariates, including baseline GEP and disease type. These primary analyses will follow the intention-to-treat principle.

In addition, a secondary analysis of the measures of GEP at 0, 6, 12, 18 and 24 months will investigate the effect of the intervention over time. This investigation is likely to use a random effects analysis, to allow for individual variation in the level of and degree of change in GEP. Our pre-specified sub-group analyses will compare the magnitude of the treatment effect on GEP between sub-groups defined by gender, baseline GEP and disease type.

Analysis of secondary outcomes will be clearly delineated from the primary analysis in any statistical reports produced. Secondary outcomes (MSFC, category rating scales, annual overall patient and treating physician assessments of efficacy) will be scored according to standard methodology and analysed in a similar way to the primary outcome. Conventional linear regression models will be used for continuous secondary outcome measures, and logistic regression or related methods for binary and categorical outcome measures.

Imaging results will be analysed using a multi-level model enabling accommodation of missing data. The incidence rates of AEs and SAEs will be summarised by treatment group. The proportion of participants discontinuing treatment will be summarised by reason and by treatment group.

#### Secondary analysis of EDSS

The analysis of EDSS will follow the established practice of using time to first progression as the primary endpoint. However, this approach is not optimal in terms of statistical power, and we will be collecting data on disease progression for the full follow-up duration of the trial allowing an analysis based on mean scores over the follow-up period.

#### Exploratory analyses

Exploratory analyses will be clearly delineated from the analysis of primary and secondary outcomes in any statistical reports. The exploratory analysis will include prognostic and predictive factor analysis of disease progression and multivariate analysis of response data.

### Ethical considerations

The ACTiMuS trial will be undertaken in compliance with the World Medical Association Declaration of Helskinki (revised version of Seoul, 2008) and international standards of Good Clinical Practice. Trial design and processes for implementation have the approval of the UK National Research Ethics Committee (NRES Committee South West – Frenchay, 12SW0358).

## Discussion

Our phase I study established the feasibility of autologous bone marrow infusion in patients with MS, provided good evidence of safety and offered preliminary suggestions of efficacy [1]. ACTiMuS will determine whether infusion of autologous marrow does indeed lead to improvements in CNS conduction in patients with progressive MS.

Our proposed trial utilises a randomised, blinded, immediate-versus-delayed therapy controlled protocol. There are two reasons for this design. Firstly, to maintain blinding all patients must believe they could be receiving active intervention, and so all must undergo marrow harvesting. It then becomes unreasonable (and arguably unacceptable) for participants not to receive therapy. This tension is resolved by treating all subjects, but establishing a control arm by comparing immediate versus delayed administration using the stepped wedge design. Secondly, there is very limited evidence as to whether any benefit associated with BM stem cells remains constant, declines or even improves over time. By comparing immediate with delayed therapy we can test whether an additional year of follow-up is associated with any differences in outcomes.

The difficulties of demonstrating neuroprotective or reparative effects over relative short periods of time in progressive MS have been well documented [21]. The GEP has been developed as a tool that, by combining multimodal evoked potential recordings to a single score, may be used to monitor the evolution of MS in individual patients. It has also been employed as a surrogate endpoint in clinical trials, and longitudinal studies in significant numbers of patients with MS (>80) have validated the GEP as a marker of the severity of CNS damage and its progression [3, 22]. Our phase I study helped support the view that electrophysiological assessment represents a sensitive and objective surrogate approach to exploring possible improvement in patients with chronic disability in MS [1] and, combined with the supportive evidence outlined above, justifies our inclusion of change in GEP as the primary outcome measure.

In our phase I trial, we elected to use a filtered preparation of whole bone marrow, rather than one or other selected, growth factor-expanded sub-populations, for several reasons. First, this mimicked the relatively unselected mononuclear cell population that proved beneficial in the first therapeutic studies in rodent demyelinating models [23, 24]. Using unselected BM cells is an increasingly common approach, successfully explored not just in experimental models of stroke [25] and spinal cord injury [26], but also clinically, in patients with myocardial infarction [27, 28], liver disease [29] and peripheral vascular disease [30], and most recently stroke [31].

All BM stem cell sub-populations are in this way included, and several may be beneficial. Multipotent mesenchymal stromal cells (MSCs) are important [32, 33], but CD34-positive haematopoetic stem cells also have reparative potential [34, 35], as do CD133-positive stem cells [36, 37]; other less well-defined sub-populations including Stro-1 positive cells [38–42] may also be valuable [43–45]. Cell selection obviously excludes the majority of cell types; but the available evidence suggests that there is no reason to exclude any specific BM stem cell sub-population; indeed in some circumstances, unfiltered cells have better reparative potential than MSCs [46].

Additionally, the repeated cell cycling and expansion to prepare purified selected MSCs (or other sub-populations) not only induces unwelcome genetic instability [47–49], but may compromise differentiation and repair capacities [50–54]. Finally, if there were benefit from non-selected, non-expanded cells, it would be far easier (and cheaper) to adopt and apply such therapies in relatively non-specialist units, with no requirement for a sterile good manufacturing practice (GMP) cell growth or selection facility.

This ’lumping not splitting’ approach creates the difficulty of obscuring which cell sub-population(s) may actually contribute to the therapeutic effects, but these questions are important opportunities rather than problems: exploring and dissecting the cells and mechanisms relevant to the therapeutic effects should create significant opportunities for developing, refining and improving this form of cell therapy in MS, and we will explore these topics in the laboratory arm of the study which will run concurrently with the clinical trial.

ACTiMuS is the first randomised clinical trial of unfractionated bone marrow-derived cell therapy (without myeloablation) in MS and, as such, represents a landmark in the development of reparative therapies for progressive MS. Furthermore, it will test the sensitivity of employing a modified cross-over protocol with GEP as the primary outcome measure in progressive MS, with potential implication for future clinical trials of reparative and neuroprotective therapies in MS.

## Trial status

Recruitment to the ACTiMuS trial commenced in March 2014 and is ongoing.

## Abbreviations

ACTiMuS: Assessment of Cellular Therapy in progressive Multiple Sclerosis
AE: adverse event
BM: bone marrow
BMCT: bone marrow cell therapy
BSAEP: brainstem auditory evoked potential
CNS: central nervous system
CXR: chest X-ray
DTI: diffusion tensor imaging
EDSS: Expanded Disability Status Scale
FBC: full blood count
FLAIR: fluid attenuated inversion recovery
fMRI: functional MRI
GEP: global evoked potential
GMP: good manufacturing practice
Hb: haemoglobin
MEP: motor evoked potential
MMEP: multimodal evoked potential
MPRAGE: Magnetisation Prepared RApid Gradient Echo
MRI: magnetic resonance imaging
MS: multiple sclerosis
MSFC: Multiple Sclerosis Functional Composite
MSIS-29: Multiple Sclerosis Impact Scale-29
NHSBT: National Health Service Blood and Transplant
OCT: optical coherence tomography
PASAT: Paced Auditory Serial Addition Test
PPMS: primary progressive multiple sclerosis
SAE: serious adverse event
SEP: sensory evoked potential
SPMS: secondary progressive multiple sclerosis
VEP: visual evoked potential
